# Chronic Pain in the Japanese Community—Prevalence, Characteristics and Impact on Quality of Life

**DOI:** 10.1371/journal.pone.0129262

**Published:** 2015-06-15

**Authors:** Shinsuke Inoue, Fumio Kobayashi, Makoto Nishihara, Young-Chang P. Arai, Tatsunori Ikemoto, Takashi Kawai, Masayuki Inoue, Tomomi Hasegawa, Takahiro Ushida

**Affiliations:** 1 Multidisciplinary Pain Center, Aichi Medical University, Yazako Karimata, Nagakuteshi, Aichi, 480–1195, Japan; 2 Department of Health and Psychosocial Medicine, Aichi Medical University, Yazako Karimata, Nagakute, Aichi, 480–1195, Japan; 3 Institute of Physical Fitness, Sports Medicine and Rehabilitation, School of Medicine, Aichi Medical University, Yazako Karimata, Nagakute, Aichi, 480–1195, Japan; Hokkaido University, JAPAN

## Abstract

**Background:**

Chronic pain is recognized as a public health problem that affects the general population physically, psychologically, and socially. However, there is little knowledge about the associated factors of chronic pain, such as the influence of weather, family structure, daily exercise, and work status.

**Objectives:**

This survey had three aims: 1) to estimate the prevalence of chronic pain in Japan, 2) to analyze these associated factors, and 3) to evaluate the social burden due to chronic pain.

**Methods:**

We conducted a cross-sectional postal survey in a sample of 6000 adults aged ≥20 years. The response rate was 43.8%.

**Results:**

The mean age of the respondents was 57.7 years (range 20–99 years); 39.3% met the criteria for chronic pain (lasting ≥3 months). Approximately a quarter of the respondents reported that their chronic pain was adversely influenced by bad weather and also oncoming bad weather. Risk factors for chronic pain, as determined by a logistic regression model, included being an older female, being unemployed, living alone, and no daily exercise. Individuals with chronic pain showed significantly lower quality of life and significantly higher psychological distress scores than those without chronic pain. The mean annual duration of absence from work of working-age respondents was 9.6 days (range 1–365 days).

**Conclusions:**

Our findings revealed that high prevalence and severity of chronic pain, associated factors, and significant impact on quality of life in the adult Japanese population. A detailed understanding of factors associated with chronic pain is essential for establishing a management strategy for primary care.

## Introduction

Chronic non-cancer pain is a common problem that substantially impairs physical and psychological health and economic well-being. A number of studies in recent years have attempted to improve understanding of the various characteristics of chronic pain, including its prevalence. Previous estimates of the prevalence of chronic pain in general populations have ranged from 7% [1] to 55% [2], but there have only been two notable surveys of chronic pain in Japan. Hattori et al. [3] found a prevalence of chronic pain of 13% in 2006 using a web-based survey. Their participants were subdivided into age groups (18–29 years, 30–49 years and ≥50 years), but the mean age of the entire group was not reported and the authors acknowledged that their use of the Internet might have excluded a greater proportion of elderly participants. Nakamura et al. [4] reported a prevalence of 15.4% in 2011 using a postal survey. Both studies used a definition of pain intensity as ≥5 on an 11-point numeric rating scale (NRS) and a pain duration of ≥6 months. This definition may be suitable for detecting the prevalence of severe dysfunctional persistent pain, but we aimed to identify the prevalence of more general chronic pain in the Japanese community, as persistent pain can cause substantial suffering and disability, even if it is mild or moderate. Therefore, we chose to use the International Association for the Study of Pain (IASP) definition of chronic pain of that “persisting continuously or intermittently for longer than 3 months”.

Although previous reports documented the clinical consequences of chronic pain, they did not explore the social consequences, such as work loss, or the negative effects of chronic pain on quality of life (QOL) and psychological well-being. To understand the various factors that may influence chronic musculoskeletal pain in a population, it is important to make comparisons within a community with similar levels of educational achievement, health awareness and social security provision, and that lives in a similar environment. A detailed understanding of the epidemiology of chronic pain is essential for efficient management of chronic pain to address its increasing social burden.

We examined the epidemiological characteristics of and influences on chronic pain in Japanese society by means of a postal survey. This cross-sectional study provided quantitative data on the prevalence and severity of various kinds of pain, the demographic characteristics of individuals with pain, the impact of pain on work, and the relationships between chronic pain, QOL and psychological distress in a community in which educational achievement, health awareness, social security provision and climate are well understood. The existence of a relationship between chronic pain and weather conditions is well known [5, 6], but few data on this phenomenon have been collected. Therefore, we also investigated the perceived influence of bad weather and cold temperature on pain in Japan.

## Methods

### Procedures and participants

We performed a postal survey in the well-defined primary health care district of Owariasahi in November 2011. Owariasahi is a highly industrialized community covering an area of 21.03 km^2^ located in the northwest of Aichi, in the center of Honshu, Japan’s main island. The community had 82,182 inhabitants (40,321 men; 41,816 women) and 33,326 households as of January 2013, according to the Japanese Basic Resident Register Network, a national registry of Japanese citizens. Distribution of demographic characteristics in the studied population, including age, male to female ratio, and composition of economy, had no notable deviation from nationwide census data of Japan [7] (S1 Table). These data were provided from the municipal government of Owariasahi with the approval of the municipal assembly as a part of a health improvement campaign. Owariasahi participated in the first Alliance for Healthy Cities in 2004, an international network supported by World Health Organization (WHO) to protect and enhance the health of city dwellers. The questionnaire was mailed to 6,000 individuals ≥20 years old. All participants were randomly selected using the Basic Resident Register Network. The study used a cross-sectional design and data were collected on 18 consecutive days.

The survey was reviewed and approved by the Owariasahi Education and Welfare Committee and the Owariasahi municipal council on September 2011.

### Questionnaire

The questionnaire collected information on age, sex, occupation, co-residence and participation in exercise. Daily exercise was divided into three categories; “daily exercise”, “1–3 times/week” and “no regular exercise”. Participants were asked about pain intensity using an 11-point NRS (0 = no pain, 10 = worst pain imaginable), pain duration, location of pain and the perceived influence of local climate on pain symptoms.

### Definition for chronic pain

Chronic pain was defined as a “yes” answer to the question, “Do you have any chronic pain lasting 3 months or more, either all the time or intermittently (excluding toothache, migraine, and menstrual pain)?” Participants who met these criteria were assigned to the chronic pain (CP) group. We defined severe chronic pain (severe CP) as persistent or regularly recurrent pain with a duration of >6 months and pain intensity on the NRS of ≥5. The severe CP group was included as a subset of the CP group.

### Impact of chronic pain

Subjective QOL was assessed on the ‘EuroQol-5 Dimensions’ scale (EQ-5D) [8], a common instrument for assessing health-related QOL (HRQOL) that was developed in Europe. This instrument contains descriptions of health status in five dimensions: ‘mobility’; ‘self-care’; ‘usual activities’; ‘pain/complaints’ and ‘anxiety/depression’. Participants are required to indicate whether they experience no, some, or serious health problems in each dimension. The combination of responses provides a description of 243 different health states, with a set of values ranging from 1 (no problem in any dimension) to −0.111 (severe problems in all five dimensions). All EQ-5D health states are assigned values on a scale between perfect health (1) and death (0), although the scoring rules permit scores <0 for extremely impaired health states. The Japanese version of the value set was developed by the Japanese EuroQol Translation Team, based on a survey of time trade-off assessments for the general population in Japan [9].

The Kessler 6-item psychological distress scale (K6) was also used [10], which consists of six questions to quantify non-specific psychological distress, with each question rated on a five-point scale. The K6 was scored using the unweighted sum of the responses, where responses ranged from “none of the time” = 0 to “all of the time” = 4. Thus, the total range of responses was 0–24. A K6 score over 5 is considered to be a risk factor for a mood disorder in the Japanese population [11].

To assess the social consequences of chronic pain, participants were asked to report the amount of time taken off work due to pain in the past year. Only data from participants 20–59 years old were included in this analysis, excluding students and unemployed persons. Overall work loss due to pain for the whole of Japan during 2012 was estimated on the basis of the 2012 annual report by the Japanese national tax agency, including number of employees, average working days and annual income.

### Statistical analyses

Data were analyzed using SPSS version 21.0 for Windows (IBM Corp., Armonk, NY, USA). Descriptive statistics were used to present the demographic characteristics of the sample, as well as occupation, family composition, daily activity, and the location, severity and duration of chronic pain.

Continuous data are reported as the mean ± standard deviation (SD) if normally distributed, and as the median and interquartile range (IQR) if not normally distributed. Analysis of variance, Student’s unpaired t-test, and the Mann–Whitney U test were used where appropriate. Categorical data are represented as n (%), and were analyzed using Fisher’s exact test.

Simultaneous logistic regression was performed to evaluate the effect of specific demographic characteristics and social factors, as well as disease variables, on pain status. The analysis produced odds ratios and their 95% confidence intervals. P values <0.05 were considered statistically significant in all analyses.

## Results

Survey forms were completed and returned by 2,701 individuals, a response rate of 45.0%. Seventy-three respondents were excluded because of missing data, reducing the final sample size to 2,628 (43.8%). The respondents consisted of 1,104 men and 1,524 women (Table 1), with a mean age of 57.7 years (range 20–99 years).

**Table 1:** Social and demographic characteristics of all respondents, and those with or without chronic pain.

	All respondents (n = 2,628)	Without chronic pain (n = 1,596)	Chronic pain (n = 1,032)	Severe chronic pain (n = 456)
M/F, n/n (%/%)	1,104/1,524 (42.0%/58.0%)	698/898 (43.7%/56.3%)	406/626 (39.3%/60.7%)	159/297 (34.9%/65.1%)
**Age (years)**				
20–30, n (%)	185 (7.0%)	144 (9.0%)	41 (4.0%)	17 (3.7%)
31–40, n (%)	374 (14.2%)	263 (16.5%)	111 (10.8%)	46 (10.1%)
41–50, n (%)	345 (13.1%)	223 (14.0%)	122 (11.8%)	54 (11.8%)
51–60, n (%)	367 (14.0%)	201 (12.6%)	166 (16.1%)	77 (16.9%)
61–70, n (%)	673 (25.6%)	408 (25.6%)	265 (25.7%)	111 (24.3%)
71–80, n (%)	506 (19.3%)	270 (16.9%)	236 (22.9%)	111 (24.3%)
81–90, n (%)	159 (6.1%)	78 (4.9%)	81 (7.8%)	34 (7.5%)
91–100, n (%)	19 (0.7%)	9 (0.6%)	10 (1.0%)	6 (1.3%)
**Occupation**				
Full-time ^a^	810 (30.8%)	535 (33.5%)	275 (26.6%)	114 (25.0%)
Primary sector	8 (1.0%)	4 (0.7%)	4 (1.5%)	2 (1.8%)
Secondary sector	274 (33.8%)	189 (35.3%)	85 (30.9%)	29 (25.4%)
Tertiary sector	528 (65.2%)	342 (63.9%)	186 (67.6%)	83 (72.8%)
Part-time	397 (15.1%)	253 (15.9%)	144 (14.0%)	57 (12.5%)
Student	34 (1.3%)	29 (1.8%)	5 (0.5%)	2 (0.4%)
Unemployed	1,349 (51.3%)	757 (47.4%)	592 (57.4%)	274 (60.1%)
Unknown	38 (1.4%)	22 (1.4%)	16 (1.6%)	9 (2.0%)
**Family composition**				
Living with ≥3 persons	1,578 (60.0%)	1,012 (63.4%)	566 (54.8%)	242 (53.1%)
Living as a couple	834 (31.7%)	478 (29.9%)	356 (34.5%)	154 (33.8%)
Living alone	216 (8.2%)	106 (6.6%)	110 (10.7%)	60 (13.2%)
**Exercise**				
Daily	622 (23.7%)	385 (24.1%)	237 (23.0%)	89 (19.5%)
1–3 days/week	1,006 (38.3%)	592 (37.1%)	414 (40.1%)	172 (37.7%)
None	942 (35.8%)	586 (36.7%)	356 (34.5%)	187 (41.0%)
Unknown	58 (2.2%)	33 (2.1%)	25 (2.4%)	8 (1.8%)
**Duration of pain**				
3–6 months	－	0 (0.0%)	284 (27.5%)	0 (0.0%)
6–12 months	－	0 (0.0%)	197 (19.1%)	111 (24.3%)
1–3 years	－	0 (0.0%)	194 (18.8%)	115 (25.2%)
>3 years	－	0 (0.0%)	357 (34.6%)	230 (50.4%)

^a^ Full-time workers were categorized as: primary (agriculture, forestry and fishery); secondary (mining, manufacturing and construction); or tertiary (service industries).

The criteria for chronic pain were met by 1,032 respondents, an incidence of 39.3% among all respondents; severe chronic pain was reported by 456 respondents, equating to a prevalence of 17.4%. Chronic pain was more common in women (41.1%) than men (36.8%; P <0.05). The questionnaire included employment status and family structure. Full time workers represented 30.8% of all respondents. More than half of all respondents (51.3%) were unemployed. 60% of respondents were living with three or more people, 8.2% of respondents lived alone (Table 1).

**Fig 1:**
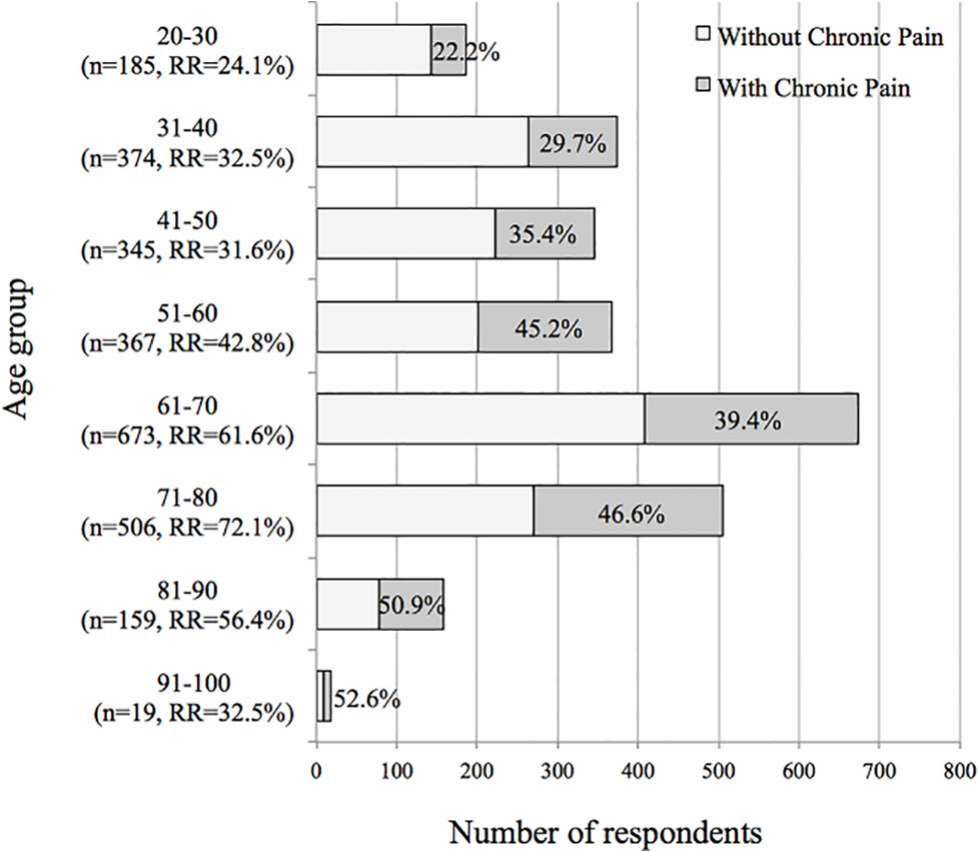
Prevalence of chronic pain by age in a Japanese population.

The prevalence of chronic pain increased with age from 22.2% to 52.6%, roughly in proportion to age, and was highest among patients in their nineties (Fig 1). The mean age of the CP group was significantly higher (60.9 ± 16.2 years) than that of the group without chronic pain (55.7 ± 17.4 years; P <0.001).

Among the 1,032 respondents with chronic pain, the mean severity on an 11-point NRS was 5.2 ± standard deviation 2.3, and 607 (58.5%) reported a pain intensity of 5 or more.

The mean severity of pain in the chronic pain (CP) group was 5.2 ± 2.3. The severe CP group had an average pain severity of 6.7 ± 1.5. The most common location of pain (one answer was allowed) was the lower back (30.6%) followed by the knees (19.8%), shoulders (17.0%) and neck (8.3%). Almost 40% of respondents had chronic spinal problems, including neck, middle back, and lower back pain, with more men reporting low back pain and more women reporting neck pain.

When asked under what conditions their chronic pain worsened or improved (Table 2), approximately 50% of respondents reported that their pain was influenced by environmental factors, with pain tending to be more intense in cold weather and less intense in warm weather. One in four respondents claimed that their pain worsened before and during bad weather (rain, snow, storms, and typhoons).

**Table 2:** Influence of activities and weather on chronic pain.

	Chronic pain			Severe chronic pain		
	Better	Worse	No Change	Better	Worse	No Change
	n (%)	n (%)	n (%)	n (%)	n (%)	n (%)
At rest	498 (62.4%)	46 (5.8%)	254 (31.8%)	214 (60.5%)	23 (6.5%)	117 (33.1%)
During activity	77 (9.9%)	378 (48.8%)	319 (41.2%)	26 (7.3%)	200 (56.3%)	129 (36.3%)
Oncoming bad weather	36 (5.1%)	168 (23.7%)	505 (71.2%)	7 (2.2%)	94 (29.8%)	214 (67.9%)
During bad weather	0.7% (5)	171 (24.9%)	512 (74.4%)	0 (0.0%)	100 (31.8%)	214 (68.2%)
Cold conditions	4.4% (33)	348 (46.9%)	361 (48.7%)	5 (1.5%)	185 (55.6%)	143 (42.9%)
Warm conditions	45.2% (327)	19 (2.6%)	378 (52.2%)	137 (43.1%)	15 (4.7%)	166 (52.2%)

Variations in the total number are a consequence of missing values.

The epidemiological data of Owariasahi city in 2011 was as follows: mean temperature 16.1°C (min -3.2°C, max 36.7), mean humidity 67%, mean annual precipitation 1,785.5 mm, mean annual air pressure 1,008.1Pa, and annual total sunshine 2,151.5 hours.

The questionnaire also obtained information about daily exercise: 23.7% of respondents reported they exercised daily, 29.5% exercised 1–3 times a week, and the remaining 44.6% did no regular exercise. The daily exercise group reported a lower frequency of severe chronic pain (14.3%) than the groups that reported exercising 1–3 times/week (17.1%) or no regular exercise (19.9%; P<0.001).

**Fig 2:**
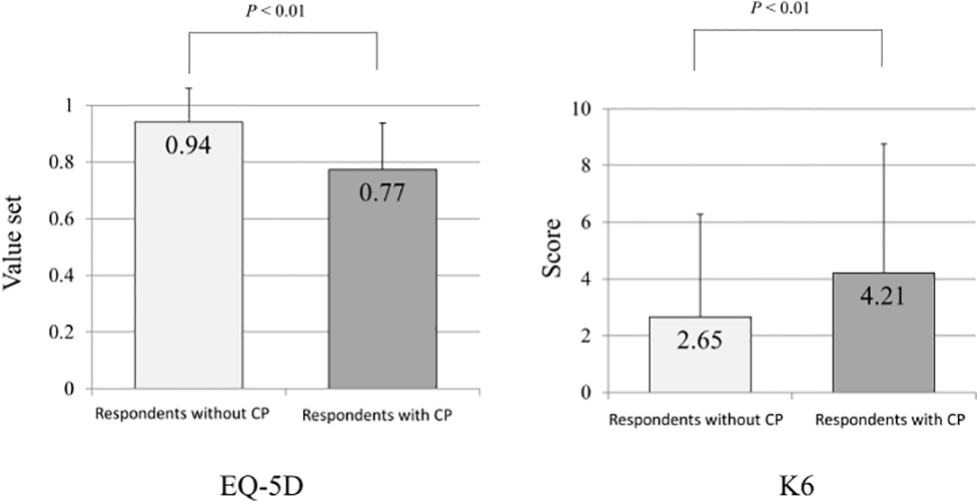
Influence of chronic pain on health-related quality of life and psychological distress. Statistical analysis undertaken with independent *t* test; mean values are shown within the columns, error bars represent standard deviation.

Individuals with chronic pain showed significantly lower utility values on the EQ-5D and higher K6 scores than those without chronic pain (Fig 2). The utility value of all respondents was 0.88 ± 0.16. When the utility value of the EQ-5D was analyzed in relation to the presence of chronic pain, the mean values were significantly lower in the CP group (0.77 compared with 0.94 for the group without chronic pain: P <0.001).

The severe CP group had very low QOL scores (0.73± 0.17) and high K6 scores; the CP group’s K6 mean was 5.2 ± 5.2, which is above the threshold of 5 for anxiety disorder. Complaints of chronic pain were more frequent in the group that did not exercise than in the group who exercised every day, and their QOL was also low (0.75 ± 0.20 compared with 0.80 ± 0.18).

Logistic regression analysis was undertaken to obtain a subset of sociodemographic variables associated with chronic pain and severe chronic pain (Table 3). The analysis found that sex, age group, occupation, family composition and daily exercise were significantly associated with both chronic pain and severe chronic pain. An asterisk indicates which group was the reference group for each variable in the regression models. More women reported chronic pain than men. Older age, living alone, lack of daily exercise and being unemployed were also associated with chronic pain.

**Table 3:** Logistic-regression modeling to identify factors associated with chronic pain.

	Chronic pain (vs. without chronic pain)			Severe chronic pain (vs. without chronic pain)		
	OR	95%CI	P-value	OR	95%CI	P-value
Female (vs, male)*	1.304	1.108–1.534	0.001	1.621	1.299–2.023	<0.001
Age (per 1 year old)	1.019	1.014–1.024	<0.001	1.022	1.015–1.029	<0.001
Age-group (years)♯						
20–30 (n)	1.000	ref		1.000	ref	
31–40 (n)	1.471	0.974–2.221	0.067	1.482	0.819–2.684	0.194
41–50 (n)	1.939	1.285–2.927	0.002	2.125	1.183–3.817	0.012
51–60 (n)	2.921	1.950–4.374	<0.001	3.368	1.907–5.950	<0.001
61–70 (n)	2.349	1.605–3.438	<0.001	2.478	1.434–4.283	0.001
71–80 (n)	3.180	2.153–4.695	<0.001	3.795	2.185–6.591	<0.001
81–90 (n)	3.833	2.401–6.120	<0.001	4.183	2.185–8.007	<0.001
91–100 (n)	4.094	1.556–10.772	0.002	6.343	1.994–20.174	0.002
Occupation†						
Full-time	1.000	ref		1.000	ref	
Part-time	0.933	0.714–1.219	0.61	0.808	0.557–1.172	0.261
Student	0.490	0.185–1.297	0.151	0.464	0.107–2.009	0.305
Unemployed	1.014	0.807–1.274	0.904	1.044	0.769–1.418	0.782
Family composition†						
Living with ≥3 persons	1.000	ref		1.000	ref	
Living in a couple	1.080	0.902–1.294	0.401	1.086	0.855–1.381	0.499
Living alone	1.442	1.067–1.947	0.017	1.763	1.221–2.547	0.003
Daily exercise†						
Daily	1.000	ref		1.000	ref	
1–3 days/week	1.237	1.004–1.525	0.046	1.384	1.034–1.853	0.029
None	1.179	0.949–1.465	0.137	1.701	1.267–2.282	<0.001
EQ-5D†						
Value (per 0.1)	0.464	0.435–0.496	<0.001	0.403	0.368–0.440	<0.001
K6†						
K6 point (per 1)	1.113	1.087–1.139	<0.001	1.158	1.126–1.191	<0.001

Abbreviations: OR: odds ratio; 95%CI, 95% confidence interval; ref, reference category.

Odds ratios were adjusted for age*, sex♯ or both†.

Finally, we analyzed absence from work caused by chronic pain. Among the 1,221 respondents aged 20–59 years (excluding unemployed persons and students), half of the workforce (52.7%) reported having chronic pain for at least 3 months. 52.7% of the workforce reported having chronic pain for at least 3 months. Two hundred twenty three respondents indicated that they had been absent from work because of pain, excluding toothache, migraine, and menstrual pain, in the previous year, for a total of 3,534 days (Table 4). The mean duration of absence from work due to pain was 9.6 days (range 1–365 days). Furthermore, a total of 4.5% of the workforce was absent from work for ≥1 week in the previous year because of their pain.

**Table 4:** Work loss in respondents reporting chronic pain.

Number of days off work	Chronic pain		Severe CP	
	Number of respondents^a^	%	Number of respondents^a^	%
1 day	61	38.1%	21	28.4%
2 days	29	18.1%	10	13.5%
3 days	13	8.1%	5	6.8%
4–6 days	14	8.8%	9	12.2%
1 week	6	3.8%	5	6.8%
~2 weeks	10	6.3%	8	10.8%
~1 month	12	7.5%	9	12.2%
~3 months	8	5.0%	4	5.4%
~1 year	7	4.4%	3	4.1%
Total	160	100.0%	74	100.0%
Days off work (mean ± standard deviation)	17.2 ± 54.0		20.5 ± 60.6	
Total days off work	2,752		1,518	

^a^Students, the unemployed, and those over the age of 60 were excluded from the analysis.

## Discussion

We determined the extent and impact of chronic pain on a general population in Japan.

### The prevalence of chronic pain

A large number of epidemiological investigations of chronic pain have recently been published (Table 5). The 21 studies we reviewed yielded a median point prevalence of chronic pain of 26% in the adult population, ranging from 7% to 55%, variation that can likely be explained by the different settings in which they were conducted. The prevalence of chronic pain reported in different studies varies a great deal, potentially being influenced by differences in survey method, country or the definition of chronic pain used. We chose to conduct a postal survey as many young people in Japan use mobile telephones instead of having a landline in their residence, and the elderly are less likely to have access to the Internet. We also chose to use the IASP definition of chronic pain: the majority of the 21 studies shown in Table 5 defined chronic pain as persistent pain for >3 months and did not take the severity of pain into account.

**Table 5:** Incidence of chronic pain reported in other global populations.

Author	Published	Country	Survey method	Participants	Response rate	Age	Prevalence	Definition of Chronic Pain	
								Duration (months)	Pain Severity
Crook J et al. [12]	1984	Canada	Telephone	827	unknown	≥18	16%	–	–
Bowsher D et al. [1]	1991	UK	Telephone	2,942	unknown	≥15	7%	3	–
Croft P et al. [13]	1993	UK	Postal	1340	75%	18–85	35%	3	–
Andersson HI et al. [2]	1993	Sweden	Postal	1806	90%	25–74	55.2%	3	–
Elliott AM et al. [14]	1999	UK	Postal	3065	82.3%	≥25	50.4%	3	–
Blyth FM et al. [15]	2001	Australia	Telephone	17,543	70.8%	≥16	18.6%	3	–
Catala E et al. [16]	2002	Spain	Telephone	5,000	42%	18–95	23.4%	3	–
Ng KF et al. [17]	2002	China	Telephone	1,051	47.7%	≥18	10.8%	3	–
Rustøen T et al. [18]	2004	Norway	Postal	1,912	48.5%	19–81	24.4%	3	–
Breivik H et al. [19]	2006	Europe	Telephone	46,394	54%	≥18	19%	6	≥5
Moulin DE et al. [20]	2007	Canada	Telephone	2,012	19.1%	18–75	29%	6	–
Neville A et al. [21]	2008	Israel	Telephone	3,738	92%	≥25	46%	3	–
Sá KN et al. [22]	2008	Brazil	Interview	2,297	97.1%	≥20	41.4%	6	–
Yeo SN et al. [23]	2009	Singapore	Telephone	4,141	43.6%	18–85	8.7%	3	≥4
Johannes CB et al. [24]	2010	USA	Internet	27,035	75.7%	≥18	30.7%	6	–
Toblin RL et al. [25]	2011	USA	Telephone	4,090	62%	≥18	26.0%	–^a^	–
Raftery MN et al. [26]	2011	Ireland	Postal	1,204	38%	≥18	36%	3	–
Nakamura M et al. [4]	2011	Japan	Postal	11,507	60%	≥18	15.4%	6	≥5
Azevedo LF et al. [27]	2012	Portugal	Telephone	5,094	76%	≥18	36.7%	3	–
Kurita, GP et al. [28]	2012	Denmark	Post or Internet	14,925	60.7%	≥16	26.8%	6	–
Shibata, M et al. [29]	2014	Japan	Interview	927	46%	≥40	47%	6	–

A dash (–) indicates no limitation.

^a^ Respondents answered “yes” to the question, “Do you suffer from any type of chronic pain, that is, pain that occurs constantly or flairs up frequently?”

According to the definition of chronic pain offered by IASP, “chronic pain is pain that persists beyond normal tissue healing time, which is assumed to be 3 months”. The median prevalence of chronic pain in the 14 studies that used this definition in adults was 29.1%, in our population it was 39.3%—suggesting that approximately 22 million people in Japan suffer from chronic pain to some extent. In contrast, Breivik et al. [19] reported the overall prevalence of chronic pain to be 19% in a large-scale computer-assisted telephone survey of 15 European countries and Israel, in which chronic pain was defined as “pain ≥5 on a 10-point NRS scale, lasting for at least 6 months, with the pain experienced within the last month, and at least twice per week”. Their criteria for chronic pain reflected that experienced by the severe CP group in our population. The prevalence of severe CP in our study was 17.4%, corresponding to the middle position of the 16 European countries in Breivik’s study.

### Low QOL and psychological distress caused by chronic pain

Those reporting chronic pain in our study were more likely to be depressed and have low QOL. Becker et al. [30] reported that health-related QOL, measured using the Medical Outcome Study-Short Form (SF-36) technique, and mood were significantly lower in 150 consecutive patients with chronic non-malignant pain referred to a Danish multidisciplinary pain center than the general Danish population. Furthermore, 58% of patients had scores indicating either depressive or anxiety disorders. Anxiety and depressive disorder have been shown to be associated with the presence or clinical course of chronic pain. Several previous surveys have shown that individuals with chronic pain in the general population are more likely to have a psychological or psychiatric disorder [31, 32, 33]. Our findings suggest that individuals with severe chronic pain had significantly lower QOL scores and significantly higher depression scores than individuals without chronic pain.

The weighting of the EQ-5D varies by country; therefore, it is necessary to compare our results to other EQ-5D studies in Japan. We found that the utility value of respondents with severe chronic pain was 0.73, which is lower than that of patients with chronic renal failure (0.798) [34] and chronic schizophrenia (0.75) [35] in Japanese QOL studies that have used EQ-5D (Table 6).

**Table 6:** EQ-5D value sets reported by clinical studies in Japan.

Value set	Status	N	Author	Published
1	Full health			
0.846	Diabetes mellitus type 2	220	Sakamaki H. et al. [36]	2006
0.808	Asthma	54	Oga T. et al. [37]	2003
0.798	Chronic renal failure	71	Tajima R. et al. [34]	2010
0.75	Chronic schizophrenia	47	Nakamae T. et al. [35]	2010
0.665	Dementia, Alzheimer type	72	Hachimori A. et al. [38]	2009
0.49	Arteriosclerosis obliterans (critical limb ischemia)	289	Aramoto H. et al. [39]	2003
0.37	Destructive spondyloarthritis with rheumatoid arthritis	25	Uehara M. et al. [40]	2012
0	Death			

The reported K6 scores also showed that there is a high burden of mood disorder among those with chronic pain: the mean K6 score in the severe CP group was 5.2; a K6 score over 5 points is considered to indicate mood disorder in the Japanese population [11]. Bair et al. also reported that among primary care patients with chronic musculoskeletal pain, comorbid depression or anxiety is strongly associated with more severe pain and greater disability [41]. These results indicate that persistent pain has an adverse association with psychological distress and QOL, and underline the need to prevent physical dysfunction and psychological disorders caused by long-lasting pain.

### Weather and pain

Many patients with chronic pain complain that their condition is aggravated by changes in the weather [5, 6]. Several studies support the view that meteorological factors (e.g. low temperature, high atmospheric pressure, high or increased relative humidity) can affect musculoskeletal pain [42] and rheumatic pain [43, 44], while others have found a weak or no association [45, 46, 47]. We found that body movement, cold conditions, and bad weather were associated with the aggravation of pain (Table 2). Conversely, warm conditions and rest were associated with amelioration of pain. Approximately 25% of respondents with chronic pain perceived that changes in the weather affected their chronic pain. Moreover, almost all the respondents who reported that their pain was affected by bad weather also reported that weather changes predicted an increase in pain. Our findings show that not only weather changes, but also impending bad weather appear to be responsible for increases in chronic pain.

### Family composition and chronic pain

Interesting relationships between chronic pain and family structure were observed. Previous studies have reported that individuals living alone, or who are divorced, have a higher prevalence of musculoskeletal pain [48, 49, 50]. We found that people living alone report more intense chronic pain than people living with three or more other people. This may be related to the radical change in the family structure of modern Japan, which is characterized by an increase in the proportion of one-person households from 18% to 25.5% between 1986 and 2010 [7]. A sharp increase in the proportion of elderly individuals living alone (from 6% to 14%) was also seen during this period. Based on these data, the number of elderly individuals suffering from loneliness is predicted to increase markedly in the future, and become a major social issue in Japanese society. Given this prediction, measures need to be taken to maintain musculoskeletal function and psychological health to facilitate participation in the wider community.

### Work loss due to chronic pain

In the United States, an average of 5.2 hours/week of productive time was lost due to musculoskeletal pain [51] and individuals with work-related pain lost 101.8 million workdays owing to back pain [52]. Another study revealed that the estimated total impact of chronic pain among 2,459 employees was approximately $US4607 per employee per year for pain-related healthcare including the medical and pharmacy costs. [53]. We found that 234 individuals missed work or housework on at least one day per year due to pain, and the mean duration of absence from work due to pain was 9.6 days. Furthermore, 55 of these individuals were absent from their work for >1 week. We calculated the economic loss due to chronic pain based on the data of mean work days [54] and the mean annual income of each sector for different age groups [55] and the population by labor force status [7]. Applying these results to the Japanese work environment, we estimate that overall work loss due to chronic pain totaled approximately ¥1,953 billion (US$19.9 billion) in Japan in 2012. When the loss of opportunity to work, a decrease in employment capacity and the burden of care for families are also taken into account, the increase in socioeconomic costs due to chronic pain is likely to be much greater.

## Limitations

The limitation of this study was the relatively low response rate (43.8%), which may have influenced the prevalence rate of chronic pain that we report. As shown in Table 5, there is a wide variety of response rates that appear to depend on the country studied or research methods used. The mean response rate of all surveys listed in Table 5 was 63 ± 20.5%, in two of the studies the response rate was below 40%. We did not offer remuneration or reward for the respondents to avoid introducing a potential response bias, but this might explain why the response rate was not higher.

We focused on the relationship between chronic pain and the climate or environmental situation in this study. We did not measure or quantify these kinds of environmental factors because the respondents did not recognize precise atmospheric pressure or temperature. However, almost half of respondents believe their pain to be related to some kind of environmental factor. Our data imply that the further investigation of sensory information from the point of view of environmental conditions may provide us with a new approach to overcoming formidable chronic pain.

## Conclusion

We found that the prevalence of chronic pain was approximately 40% in a general Japanese population. Chronic pain has a significant impact on occupational and daily social life, and seriously affects psychological health. As the population of Japan ages, it is important to recognize that chronic pain is a serious social issue, which should be addressed by the entire Japanese society. Specific and effective interventions are needed to reduce the prevalence of musculoskeletal pain and its debilitating effects. Apart from the physical disabilities associated with chronic pain, our findings show that chronic pain is associated with mental health issues, decreased QOL and social loss due to absence from work. Our data provide a scientific basis for estimating the burden of chronic pain in Japanese communities. National countermeasures are required to address chronic pain, including the promotion of daily exercise habits to address persistent musculoskeletal pain.

## Supporting Information

S1 Table The comparison of average demographical data between Owariasahi and Japan in 2012. (TIF)
