# Supplementary figures and images for: Chronic Pain in the Japanese Community—Prevalence, Characteristics and Impact on Quality of Life
Source: PLoS One. 2015 Jun 15;10(6):e0129262. doi: 10.1371/journal.pone.0129262 (PMC4467865; doi:10.1371/journal.pone.0129262)

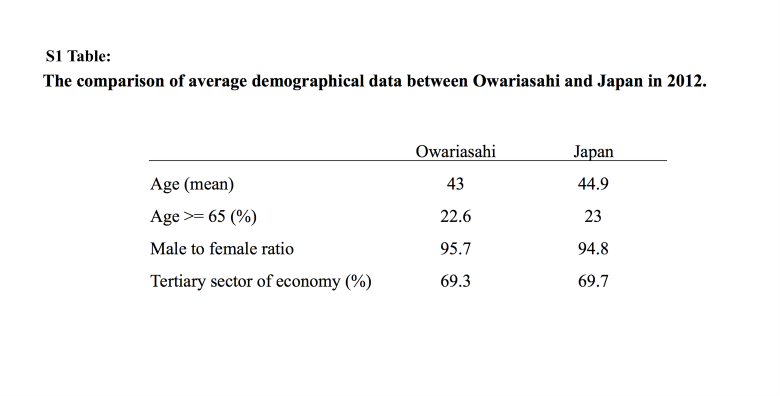

Supplement: S1 Table — (TIF) [file pone.0129262.s001.tif]
